# Genetic and phenotypic effects of chromosome segments introgressed from *Gossypium barbadense* into *Gossypium hirsutum*

**DOI:** 10.1371/journal.pone.0184882

**Published:** 2017-09-20

**Authors:** Weiwu Song, Mi Wang, Wei Su, Quanwei Lu, Xianghui Xiao, Juan Cai, Zhen Zhang, Shaoqi Li, Pengtao Li, Juwu Gong, Wankui Gong, Haihong Shang, Aiying Liu, Junwen Li, Tingting Chen, Qun Ge, Yuzhen Shi, Youlu Yuan

**Affiliations:** 1 State Key Laboratory of Cotton Biology, Key Laboratory of Biological and Genetic Breeding of Cotton, The Ministry of Agriculture, Institute of Cotton Research, Chinese Academy of Agricultural Sciences. Anyang, Henan, China; 2 Enshi Academy of Agricultural Sciences. Enshi, Hubei, China; 3 College of Agriculture, Yangtze University, Jingzhou, Hubei, China; Fujian Agriculture and Forestry University, CHINA

## Abstract

MBI9915 is an introgression cotton line with excellent fiber quality. It was obtained by advanced backcrossing and continuous inbreeding from an interspecific cross between the upland cotton (*Gossypium hirsutum*) cultivar CCRI36 as the recurrent parent and the sea island cotton (*G*. *barbadense*) cultivar Hai1, as the donor parent. To study the genetic effects of the introgressed chromosome segments in *G*. *hirsutum*, an F_2_ secondary segregating population of 1537 individuals was created by crossing MBI9915 and CCRI36, and an F_2:3_ population was created by randomly selecting 347 individuals from the F_2_ generation. Quantitative trait locus (QTL) mapping and interaction for fiber length and strength were identified using IciMapping software. The genotype analysis showed that the recovery rate for MBI9915 was 97.9%, with a total 6 heterozygous segments and 13 homozygous segments. A total of 18 QTLs for fiber quality and 6 QTLs for yield related traits were detected using the two segregating generations. These QTLs were distributed across 7 chromosomes and collectively explained 0.81%–9.51% of the observed phenotypic variations. Six QTLs were consistently detected in two generations and 6 QTLs were identified in previous studies. A total of 13 pairs of interaction for fiber length and 13 pairs of interaction for fiber strength were identified in two generations. Among them, 3 pairs of interaction for fiber length and 3 pairs of interaction for fiber strength could be identified in all generations; 4 pairs of interactions affected fiber length and fiber strength simultaneously. The results clearly showed that 5 chromosome segments (Seg-5-1, Seg-7-1, Seg-8-1, Seg-20-2 and Seg-20-3) have important effects on fiber yield and quality. This study provides the useful information for gene cloning and marker-assisted breeding for excellent fiber related quality.

## Introduction

Cotton is the most important natural fiber in the textile industry. With improved living standards and the development of the textile industry, higher quality cotton fiber is desirable. Upland cotton and sea island cotton are cultivated tetraploid varieties in the genus *Gossypium* [1]. Upland cotton (*Gossypium hirsutum L*.) has a high yield and wide adaptability, but relatively low fiber quality while sea island cotton (*Gossypium barbadense L*.) has low yield and limited adaptability but excellent fiber quality [2]. One approach for improving both cotton fiber quality and yield is by integrating the high yield genes of *G*. *hirsutum* and excellent fiber quality genes of *G*. *barbadense* through hybridization.

Cotton yield and quality are quantitative traits controlled by multiple genes subject to environment influences [3–5]. The use of molecular markers makes it easier for breeders to more rapidly and precisely improve economic and agronomic traits of crops [6]. Reinisch et al. [7] constructed the first cotton molecular genetic map of RFLP markers in 1994. Jiang et al. [8] detected three fiber strength quantitative trait loci (QTL) in an F_2_ generation established by the cross of *G*. *barbadense* and *G*. *hirsutum*. These explained 30.9% of the total phenotypic variation. Paterson et al. [9] detected 68 QTL related to fiber quality in multiple environments. Wu et al. [10] found 13 fiber quality trait QTL in a F_2_ population derived from hybridization of *G*. *hirsutum* Handan 208 and *G*. *barbadense* Pima 90. Jamshed et al. [11] identified 165 QTLs for fiber quality traits in a *G*. *hirsutum* recombinant inbred line. Of these, 47 QTL were stable across multiple environments. Wang et al. [12] detected 64 QTL for fiber quality and 70 QTL for yield components in a population of 178 recombinant inbred lines (RILs). Yang et al. [13] detected 44 fiber quality QTL on 17 chromosomes in BC_1_ and its derived BC_1_ F_2_ lines. In spite of these QTL data, the complex genetic background of the study populations makes QTL results difficult to use for cultivar trait improvements.

Chromosome segment substitution lines (CSSLs), also known as introgression lines, are permanent populations that possess the same genetic background as the recurrent parent. Differences among CSSLs usually involve only one or a few of the introgressed chromosome segments, which effectively eliminates interference from the genetic background. Therefore, CSSLs are ideal materials for QTL fine-mapping, gene cloning, and study of QTL interactions [14]. Since Eshed and Zamir first constructed introgression lines of tomato [15], CSSLs have been successfully applied in rice [16], corn [17], and wheat [18]. However, CSSLs are less commonly used for cotton QTL mapping. Stelly et al. [19] constructed 17 chromosome substitution lines of *G*. *barbadense* in the TM-1 background of *G*. *hirsutum*. Luan et al. [20] detected 24 QTL associated with fiber yield and quality using two *G*. *hirsutum*. introgression populations. Zhu et al. [21] detected 2 QTL for lint percent and a QTL for seed index in F_2_ and F_2:3_ populations derived from a cross between two introgressed lines. Wang et al. [22] identified six stable QTL associated with fiber quality using 174 introgression lines. Cao et al. [23] fine-mapped clustered QTL for fiber quality on chromosome 7 using a *G*. *barbadense* introgressed line. Wang et al. [24] detected 24 QTL for fiber quality and lint quantity based on three phenotypic datasets collected over 2 years in two locations.

To introgress the preferred fiber quality from *G*. *barbadense* into a commercial *G*. *hirsutum* variety, a high-density simple sequence repeat (SSR) genetic linkage map was developed from a BC_1_ F_1_ population derived from an interspecific backcross between the highly resistant line Hai1 (*G*. *barbadense*) and CCRI36 (*G*. *hirsutum*) as the recurrent parent [25]. A total of 48 QTLs for verticillium wilt resistance were identified in BC_1_ F_1_, BC_1_ S_1_ and BC_2_ F_1_ populations from the same parents [26]. A total of 20 QTL for yield traits and 33 QTL for fiber quality traits were detected using 303 chromosome segment substitution lines (BC_5_ F_2_) [27]. Genetic effects and heterosis of yield and yield component traits were analyzed through hybridizing 10 chromosome segment substitution lines (CSSLs) each from two CSSL populations that produced 50 F_1_ hybrids according to North Carolina Design II [28]. Chromosome segment substitution lines MBI9804, MBI9855, MBI9752 and MBI9134 were used to construct a multiple parent population of (MBI9804×MBI9855)×(MBI9752×MBI9134). A total of 24 QTLs controlling fiber quality and 11 QTLs controlling yield traits were detected using the three segregating generations of double-crossed F_1_ and F_2_ and F_2:3_ [29].

We focused on the genetic effects of the introgressed segments in the introgression line with excellent fiber quality, MBI9915, which was selected from the BC_5_ F_3:5_ of an interspecific cross between CCRI36, a cultivar of *G*. *hirsutum* as the recurrent parent, and Hai1, a cultivar of *G*. *barbadense*) as the donor parent. An F_2_ secondary segregating population was constructed by crossing CCRI36 and MBI9915. A F_2:3_ generation was constituted by random selection of 347 individuals from the F_2_ population. QTL mapping and interaction for fiber length and fiber strength were identified by SSR markers.

## Materials and methods

### Materials

The introgression line with excellent fiber quality, MBI9915, was selected from the BC_5_ F_3:5_ of an interspecific cross of *G*. *hirsutum* CCRl36 (Chinese Cotton Research Institute 36) as the recipient parent and *G*. *barbadense* Hai 1 as the donor parent [30]. The line was used to produce an F_2_ secondary segregating generation including 1537 individuals with the other parent of CCRI36, and the F_2:3_ generation was formed by randomly selecting 347 F_2_ individuals.

In 2013, the F_2_ generation was planted in 68 rows at the Institute of Cotton Research of Chinese Academy of Agricultural Sciences (Anyang, Henan Province). The parental lines and F_1_were planted in two rows, respectively. In 2014, a total of 347 F_2_ individual plants were randomly selected as F_2:3_ rows of plants, and CCRI36 was planted as the control in one row for 20 experimental rows in the experimental farm(Anyang, Henan Province).Each row was 5 m long and 0.8 m apart with about 20 plants in each year.

### Investigation of fiber yield and quality traits

In 2013, the phenotypic traits of individual plants were studied. Naturally opened bolls were harvested and evaluated for boll weight (BW), lint percentage (LP), fiber length (FL), fiber micronaire (FM), and fiber strength (FS). In 2014, 30 naturally opened bolls in each plot were harvested for evaluation as in 2013. The fiber quality traits were tested with an HFT9000 in the Cotton Quality Supervision and Testing Center of the Ministry of Agriculture of China. HVICC international calibration cotton samples were used.

### DNA extraction and SSR molecular detection

Young leaves of the parental lines, F_1_, and F_2_ individual plants were sampled. DNA was extracted using a modified CTAB method [31]. SSR amplification and polyacrylamide gel electrophoresis were performed following the method of Zhang [32].

All the SSR markers in the genetic linkage map constructed by Shi [25] were used for screening of polymorphisms among the parental lines. A total of 41 markers were identified to be polymorphic for genotyping the F_2_ individual plants. The sequences of the SSR primers were uploaded to the CMD database (http://www.cottonmarker.org/). The primers used in the present study were synthesized by Beijing Sun biotech Co., Ltd. (Beijing, China).

### Data analysis

SAS 9.2 software was used for the descriptive statistical analysis and correlation analysis of fiber quality traits (including FL, FS and FM) and yield related traits (including BW and LP) for the F_2_ individual plants and the F_2:3_ family lines.

Genotypic analysis of the parental lines and population was performed based on the SSR polymorphic results using the GGT 2.0 software developed by van Berloo (http://www.plantbreeding.wur.nl/UK/software_ggt.html) [33].

QTL mapping and interaction was performed using the QTL IciMappingV4.0 software developed by Wang et al. [34].

The nomenclature of QTL was: q + trait abbreviation + chromosome number + serial number of QTL. For example, qFL-6-10 represented the 10th QTL controlling fiber length on chromosome 6 (Chr6).

## Results

### Fiber quality and yield analysis

Mean values of MBI9915 and recurrent parent (CCRI 36) are shown in Table 1. Compared to CCRI36, MBI9915 had longer fiber (> 30 mm), stronger fiber (> 33 cN•tex^-1^) and lower micronaire. These results indicate that the MBI9915 introgression line had excellent fiber quality.

**Table 1:** Phenotypic data of fiber quality and yield traits for parents.

	Year	BW (g)	LP (%)	FL (mm)	FM (unit)	FS (cN/tex)
MBI9915	2014	5.36±0.20	39.90±1.41	32.08±0.86	4.18±0.20	35.78±1.64
	2013	5.06±0.23	36.54±1.36	31.46±1.10	4.14±0.34	34.20±1.28
	2012	4.80±0.35	35.80±1.21	31.70±0.68	3.83±0.26	32.89±0.58
CCRI36	2014	5.48±0.35	39.47±0.81	29.28±0.88	4.49±0.17	31.29±0.88
	2013	5.33±0.25	37.44±0.73	28.93±1.08	4.37±0.10	30.31±0.79
	2012	4.98±0.26	36.22±0.87	28.99±0.88	4.22±0.19	29.47±0.99

**Fig 1:**
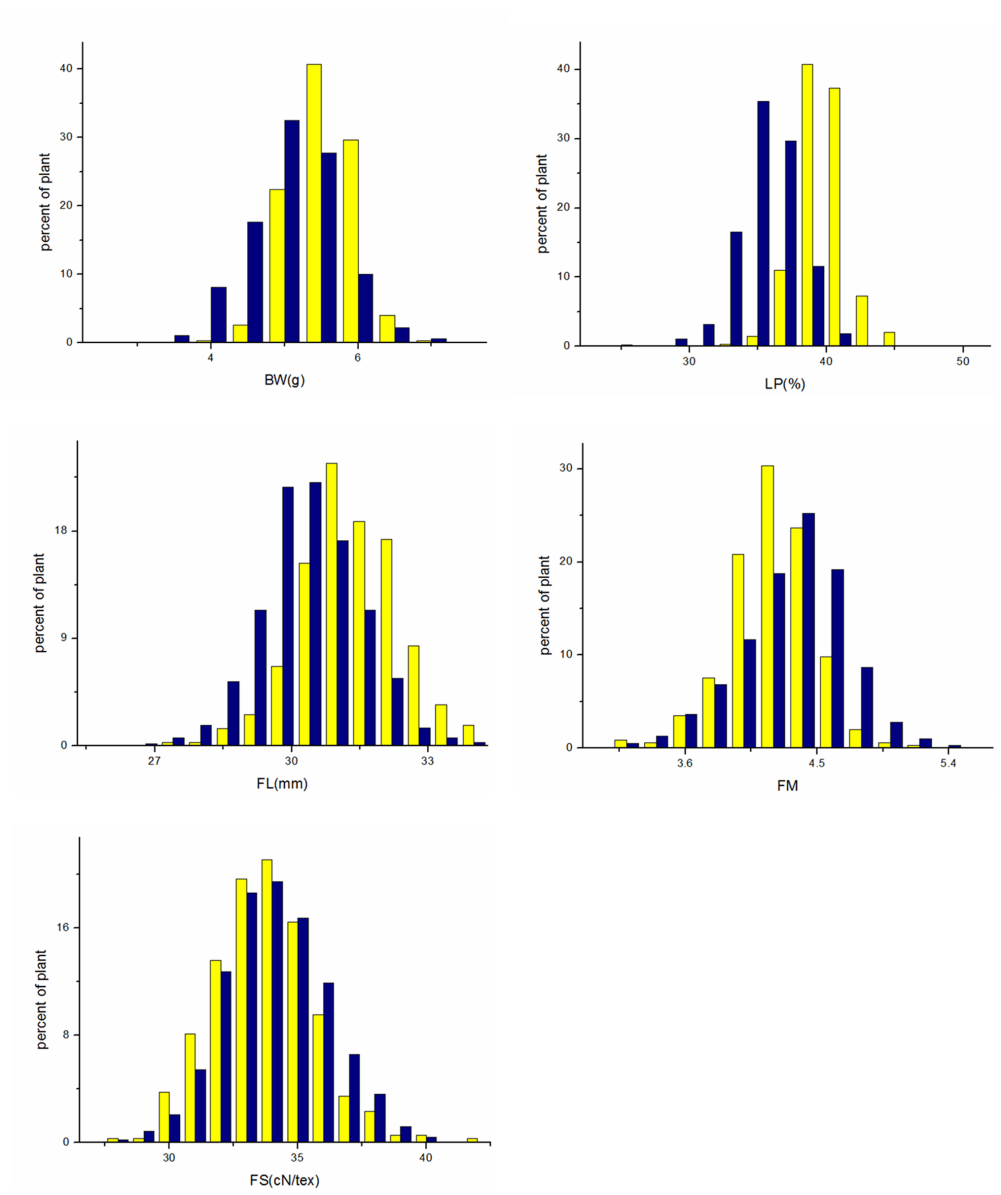
Fiber quality and yield traits of two cotton generations. Note: F_2_ generation is designated with blue square F_2:3_ generation is designated with yellow square.

The fiber quality performance and the yield related traits for the F_2_ and F_2:3_ generations are presented in Table 2 and Fig 1. The transgressive rate of F_2_ and F_2:3_ generations was 31.23%-93.94%. The variation coefficients of fiber quality and yield traits for F_2_ and F_2:3_ generations were 3.48% to 12.64%, indicating that the traits were significantly separated in the F_2_ and F_2:3_ generations. The absolute value of the skewness was < 1, indicating that the fiber quality and yield traits had a normal distribution in the two generations.

**Table 2:** Phenotypic fiber quality and yields related to the F2 and F2:3 populations.

Trait	Pop	Mean	Range	SD	Transgressive rate / %	CV / %	Skew	Kurt
BW/ g	F_2_	4.85	3.05–6.17	0.61	31.23	12.64	-0.04	0.33
	F_2:3_	5.30	3.85–6.64	0.44	53.60	8.36	-0.22	0.05
LP / %	F_2_	35.70	25.25–44.99	2.30	73.58	6.43	0.10	3.50
	F_2:3_	33.91	33.72–44.91	1.75	46.97	5.16	-0.07	0.63
FL / mm	F_2_	30.05	26.47–33.66	1.09	88.68	3.62	-0.02	0.40
	F_2:3_	31.02	27.02–33.78	1.08	93.94	3.48	-0.12	0.32
FM	F_2_	4.23	3.02–5.44	0.37	56.21	8.63	-0.10	1.31
	F_2:3_	4.10	3.03–5.02	0.28	73.49	6.83	-0.32	1.15
FS(cN·tex-1)	F_2_	33.54	27.10–41.70	2.00	90.70	5.96	0.17	0.15
	F_2:3_	33.21	27.83–41.06	1.96	87.32	5.90	0.42	0.76

### Traits correlation analysis

Correlation analysis results between fiber quality and yield related traits are presented in Table 3. The significant negative correlation between boll weight and lint percent indicates the difficulty in simultaneously improving the BW and LP. Among the fiber quality traits, fiber length and fiber strength were significantly positively correlated in the two generations, while significantly negatively correlated with fiber micronaire. This indicated that fiber quality traits are easier to simultaneously improve. Lint percent was positively correlated with fiber micronaire in the two generations, while negatively correlated with FS and FL. These results indicated the difficulty in simultaneously improving LP and fiber quality traits.

**Table 3:** Correlation coefficients among fiber quality and yield related traits in two generation.

**Traits**	**Population**	**BW**	**LP**	**FL**	**FM**
LP	F_2_	-0.189**			
	F_2:, 3_	-0.213**			
FL	F_2_	0.098**	-0.137**		
	F_2:, 3_	0.041	-0.356**		
FM	F_2_	0.310**	0.093**	-0.264**	
	F_2:, 3_	0.237**	0.330**	-0.499**	
FS	F_2_	0.005	-0.345**	0.369**	-0.178**
	F_2:, 3_	-0.058	-0.183**	0.259**	-0.094

**Significant at 0.01

### Genotypic analysis of the parents

**Fig 2:**
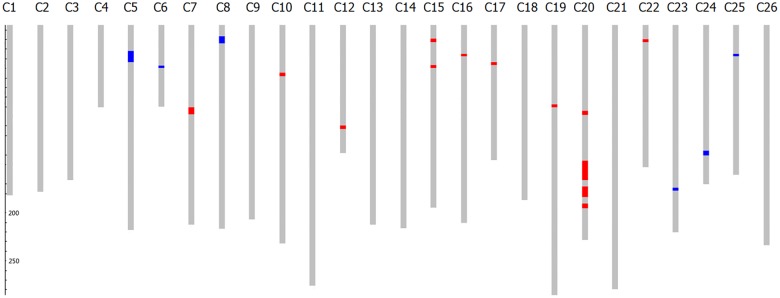
Graphical genotypes for MBI9915. Note: the genetic background of CCRI36 is designated with gray square; the heterozygous substituted segments of Hai1 is designated with blue square; the homozygous substituted segment s of Hai1 is designated with red square.

A total of 41 pairs of SSR markers were identified as polymorphic between the two parents. Introgressed Hai1 chromosome segments in MBI9915 were identified by SSR markers using GGT2.0 software (Fig 2). The background recovery rate for MBI9915 was 97.30%, with 19 *G*. *barbadense* introgression segments distributed on 15 chromosomes with a total of 105.5 cM. Among them, 13 homozygous fragments were 74.8 cM and accounted for 1.50%; 6 heterozygous fragments were 30.7 cM and accounted for 0.60%. There are 2 homozygous segments of *G*. *barbadense* on chromosome 15 and 4 homozygous segments on chromosome 20. Except for the segments on chromosomes 5, 6, 8, 23, 24 and 25, all of the others were homozygous segments.

### QTL mapping

**Fig 3:**
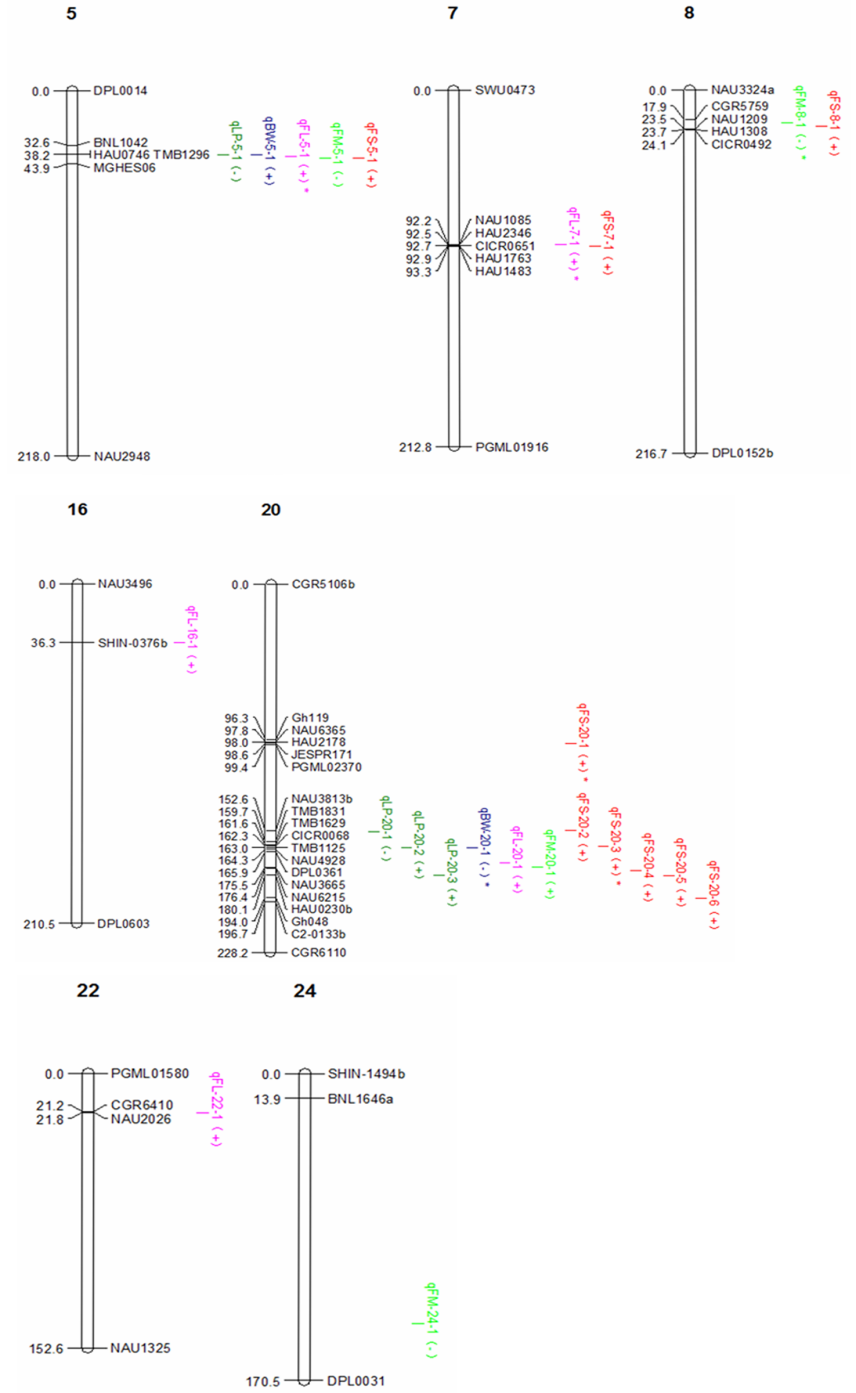
QTL of fiber quality and yield related traits mapped in the linkage map. Note: (+) indicates a positive additive effect; * indicates that the QTL could be identified in the two populations.

A total of 24 QTLs for fiber yield and quality traits were identified in F_2_ and F_2:3_, including 18 QTLs controlling four fiber quality traits and 6 QTLs controlling two yield traits, explaining 0.81%-9.51%of phenotypic variation respectively (Table 4, Fig 3). Six QTLs(qFL-5-1, qFL-7-1, qFM-8-1, qBW-20-1, qFS-20-1 and qFS-20-3) could be identified in two generations.

**Table 4:** QTL for fiber quality and yield related traits in different generation using QTL Icimapping.

Trait	QTL	Position	Pop	Chr	Nearest Marker	LOD	PVE(%)	Add	Dom
LP	qLP-5-1	38.56	F_2_	5	TMB1296	19.84	6.91	-0.85	0.16
	qLP-20-1	153.25	F_2_	20	NAU3813b	3.69	1.19	-0.04	-0.5
	qLP-20-2	163.25	F_2_	20	TMB1125	26.88	8.91	0.01	1.38
	qLP-20-3	180.25	F_2_	20	HAU0230b	3.74	1.16	0.29	-0.23
BW	qBW-5-1	38.56	F_2_	5	TMB1296	3.52	1.18	0.09	-0.04
	qBW-20-1	163.25	F_2_	20	TMB1125	7.76	2.42	-0.13	-0.01
		165.25	F_2:3_	20	TMB1125	2.57	3.51	-0.11	0.05
FL	qFL-5-1	39.56	F_2_	5	TMB1296	13.33	5.13	0.37	0.05
		38.17	F_2:3_	5	TMB1296	5.58	3.22	0.37	0.01
	qFL-7-1	92.24	F_2_	7	NAU1085	2.88	0.81	0.13	0.01
		92.24	F_2:3_	7	NAU1085	2.99	3.24	0.26	0.04
	qFL-16-1	36.31	F_2_	16	SHIN-0376b	3.25	0.92	0.12	0.13
	qFL-20-1	172.25	F_2_	20	NAU3665	7.98	2.42	0.24	0.02
	qFL-22-1	21.84	F_2:3_	22	NAU2026	3.01	3.25	0.27	-0.07
FM	qFM-5-1	40.56	F_2_	5	TMB1296	11.87	4.82	-0.10	0.06
	qFM-8-1	19.86	F_2_	8	NAU1209	20.96	6.37	-0.13	0.02
		23.86	F_2:3_	8	NAU1209	4.69	3.47	-0.08	0.08
	qFM-20-1	175.25	F_2_	20	NAU3665	19.61	5.44	0.12	0.00
	qFM-24-1	138.96	F_2:3_	24	BNL1646a	4.39	3.26	-0.08	0.05
FS	qFS-5-1	40.56	F_2_	5	TMB1296	8.07	3.38	0.55	0.06
	qFS-7-1	93.24	F_2_	7	NAU1085	19.36	5.97	0.23	0.88
	qFS-8-1	21.86	F_2_	8	NAU1209	7.00	2.12	0.41	-0.10
	qFS-20-1	98.25	F_2_	20	Gh119	31.20	9.13	0.92	-0.03
		96.25	F_2:3_	20	Gh119	3.06	1.69	0.44	-0.36
	qFS-20-2	152.25	F_2_	20	NAU3813b	4.54	1.26	0.07	0.44
	qFS-20-3	162.25	F_2_	20	TMB1125	16.57	4.75	0.06	0.88
		162.96	F_2:3_	20	TMB1125	3.02	1.66	0.52	-0.14
	qFS-20-4	177.25	F_2_	20	NAU6215	18.30	5.44	0.05	-0.92
	qFS-20-5	180.25	F_2_	20	HAU0230b	18.30	5.21	0.03	0.92
	qFS-20-6	194.00	F_2:3_	20	Gh048	3.21	1.77	0.52	-0.17

Boll weight: Two QTLs for BW were mapped on Chr05 and Chr20, explaining 1.18%–3.51% of the observed phenotypic variations. qBW-20-1 was identified in two generations, linked to the marker TMB1125, and the additive effect was -0.13 and -0.11, respectively. The negative additive effect indicated that CCRI36 alleles increased boll weight. qBW-5-1 was identified on chromosome 5, and the positive additive effect indicated that Hai1 alleles increased boll weight.

Lint percent: A total of 4 QTLs for LP were identified. One was mapped on chromosome 5 and three were mapped on chromosome 20, explaining 1.16%~8.91% of the observed phenotypic variation. The negative additive effect for qLP-5-1 and qLP-20-1 indicated that the CCRI36 alleles increased the lint percent. qLP-20-2 and qLP-20-3 had the opposite additive effect.

Fiber length: A total of 5 QTLs controlling FL were detected on five chromosomes (Chr5, Chr7, Chr16, Chr20 and Chr22), explaining 0.81%~5.13%of the observed phenotypic variations. Among them, qFL-5-1 and qFL-7-1 were identified in two generations. qFL-5-1 linked to TMB1296 and could explain 5.13% and 3.22% of the observed phenotypic variations in the F_2_ and F_2:3_ generation, respectively, with the additive effect of 0.37 mm in two generations. qFL-7-1linked to NAU1085 could explain 0.81% and 3.24%of the observed phenotypic variations in the F_2_ and F_2:3_ generations, respectively, with the additive effect of 0.13 mm and 0.26 mm in two generations, respectively. The positive additive effect for all the five QTLs indicated that Hai1 alleles increased fiber length.

Fiber strength: A total of 9 QTLs controlling FS were detected on four chromosomes (Chr5, Chr 7, Chr8 and Chr20), explaining 1.26%~9.13%of the observed phenotypic variations. Three chromosomes (Chr5, Chr7 and Chr8) had only one QTL. A total of 6 QTL, of which two QTL (qFS-20-1 and qFS-20-3) were detected in two generations, were mapped on chromosome 20. qFS-20-1linked to Gh119, explained 9.13% and 1.69% of the observed phenotypic variations, with the additive effect of 0.92 and 0.44 in F_2_ and F_2:3_, respectively. qFS-20-3 linked to TMB1125, could explain 4.75% and 1.66% of the observed phenotypic variations, with the additive effect of 0.06 and 0.52 in F_2_ and F_2:3_, respectively. The positive additive effect of all the 9 QTLs indicated that Hai1 alleles increased fiber strength.

Micronaire: A total of 4 QTLs controlling FM were detected on four chromosomes (Chr5, Chr8, Chr20 and Chr24), explaining 3.26%~6.37% of the observed phenotypic variations. Among them, qFM-8-1linked to NAU1209, explaining 6.37% and 3.47% of the observed phenotypic variations, with the additive effect was -0.13 and -0.08 in F_2_ and F_2:3_, respectively. Except for the positive additive effect of qFM-20-1, the negative additive effect for all the other QTL indicated that Hai1 alleles decreased fiber micronaire.

### Interactions between fiber length and fiber strength

Interaction between fiber length and fiber strength in the F_2_ and F_2:3_ populations were identified by Icimapping software. The nomenclature of introgression segment was: Seg + chromosome number + serial number of segment. For example, Seg-20-3 represented the 3th segment on chromosome 20 (Chr20).

**Fig 4:**
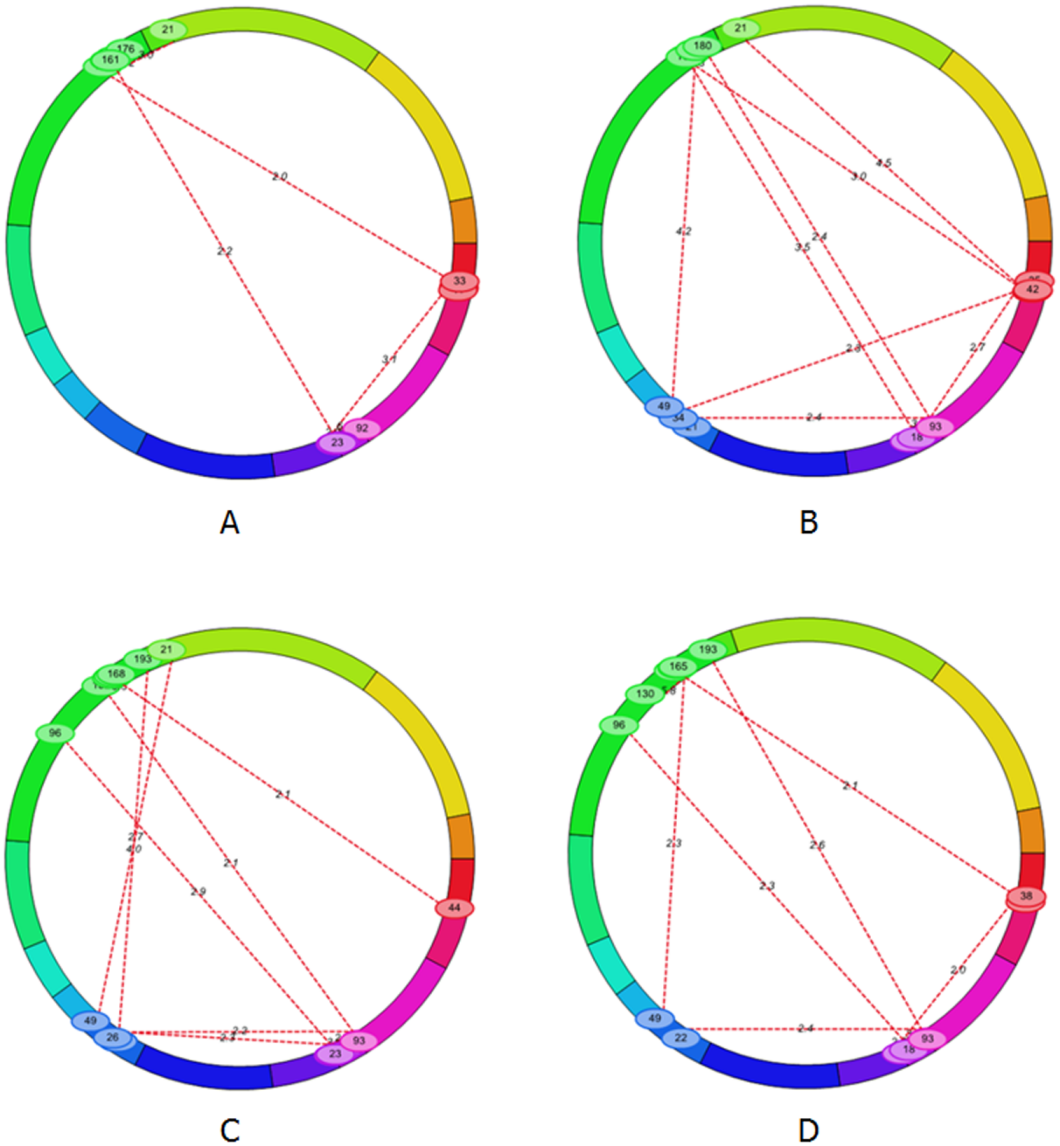
Interaction of fiber length and fiber strength in two generation. Note:the Chr5, Chr6, Chr7, Chr8, Chr10, Chr12, Chr15, Chr16, Chr17, Chr19, Chr20, Chr22, Chr23, Chr24 and Chr25 are designated with 15 kinds of colored squares from red to orange corresponding; the data on red line is LOD; the data on circle line is location on linkage map A: Interaction of FL (F2); B: Interaction of FL (F2:3); C: Interaction of FS (F2); D: Interaction of FS (F2:3).

A total of 13 pairs of interactions for fiber strength were identified in two generations, explaining 0.55%-7.60% of the observed phenotypic variations (Fig 4, S1 Table). Among these, 3 pairs of interaction (Seg-5-1 with Seg-20-2, Seg-7-1 with Seg-8-1, Seg-7-1 with Seg-15-1) for fiber strength could be identified in two generations. The main positive effects of the interaction between Seg-5-1 and Seg-20-2 were Add by Add and Dom by Add, which indicated that interaction increased fiber strength. The Add by Add effect of Seg-7-1 with Seg-8-1 was 0.30 and 0.47 in F_2_ and F_2:3_ population, respectively, which indicated that interaction increased fiber strength. The Add by Add effect of Seg-7-1 with Seg-15-1 were -0.17 and -0.37 in F_2_ and F_2:3_ populations, respectively, which indicated that interaction decreased fiber strength. The region around 17.86 cM and 22.86 cM on Seg-8-1 had the opposite interaction effect of Add by Add with Seg-20-1. The result indicated that linkage drag on Seg-8-1 should be broken.

A total of 13 pairs of interaction for fiber length were identified in two generations, explaining 0.38%-5.64%of the observed phenotypic variations (Fig 4, S2 Table). Among these, 3 pairs of interaction (Seg-7-1with Seg-8-1, Seg-8-1 with Seg-20-2, Seg-20-2 with Seg-20-3) for fiber length could be identified in the two generations. The Add by Add effects of Seg-7-1 with Seg-8-1 were 0.25 and 0.32 in F_2_ and F_2:3_ populations, respectively, which indicated that interaction increased fiber length. The main positive effects of the interaction between Seg-8-1 and Seg-20-2 were Dom by Dom. The Add by Add effect of Seg-20-2 with Seg-20-3 was 0.64 and 0.20 in F_2_ and F_2:3_ population, respectively.

A total of 4 pairs of interactions (Seg-5-1 with region around 165 cM on Seg-20-2, Seg-7-1 with Seg-8-1, Seg-7-1 with Seg-15-1, and Seg-15-2 with Seg-20-2) affected fiber length and fiber strength simultaneously.

## Discussion

### Selection of parental materials

Cotton fiber yield and quality are quantitative traits controlled by multiple genes, which are vulnerable to environmental influences [3–5]. The development of molecular markers provides crop breeders with a rapid and precise alternative approach for improving economic and agronomic traits [6]. Chromosome segment substitution lines (CSSLs) are permanent populations that possess the same genetic background as the recurrent parent, which effectively eliminates the interference of the genetic background. Therefore, CSSLs are ideal materials for QTL fine-mapping, gene cloning, and investigating QTL interactions [13].

MBI9915 was a introgression line with excellent fiber quality. It was obtained by advanced backcrossing and continuously inbreeding from an initial interspecific cross between *G*. *hirsutum* cultivar CCRI36, as the recurrent parent, and *G*. *barbadense* cultivar Hai1as the donor parent. The background recovery rate for MBI9915 was 97.30%, with 19 Hai1 introgression segments distributed on 15 chromosomes with coverage of a total 105.5 cMin, which can effectively reduce the interference of the genetic background. MBI9915 is very important research material with fiber length more than 30 mm and fiber strength more than 33 cN·tex^-1^in a three-year continuous evaluation. The research for genetic effect of the introgressed segments provides a basis for fine-mapping and cloning fiber quality QTLs.

### The stable QTL

A total of 24 QTLs for the fiber yield and fiber quality traits were identified in the F_2_ and F_2:3_ generations. Among them, qFL-5-1(linked to TMB1296), qFL-7-1(linked to NAU1085), qFM-8-1(linked to NAU1209), qBW-20-1(linked to TMB1125), qFS-20-1(linked to Gh119) and qFS-20-3(linked to TMB1125) could be identified in two generations. Six QTLs (qFL-7-1, qFS-7-1, qFS-8-1, qFL-20-1, qFS-20-2 and qFL-22-1) detected in the present study were reported in previous studies.

Fiber length: qFL-7-1 linked to NAU1085 explained 0.81% and 3.24% of the observed phenotypic variations in the F_2_ and F_2:3_ generations, with the additive effect of 0.13 and 0.26 in two generations. This QTL was reported by Ma [35] and Guo [36]. qFL-20-1 linked to NAU3665 was detected with the additive effect of 0.24, which was reported by Zhai [29]. qFL-22-1 was detected linked to NAU2026 with the additive effect of 0.27. This was also reported by Liang [26]. The result indicated that these QTL were stable between generations and in different environmental conditions.

Fiber strength: qFS-7-1 was detected on chromosome 7 with the common marker of NAU1085 reported by He [37]. qFS-8-1 was identified linked to the common marker of NAU1209 and qFS-20-2 linked to NAU3813b as reported by Zhai et al. [29].

A total of 5 stable QTL (qFL-5-1, qFM-8-1, qFS-20-1, qBW20-1 and qFS-20-3 were detected for the first time in this study.

It is difficult to detect stable QTL in different generations, different backgrounds and different environments because the quantitative traits are usually susceptible to environmental effects. Therefore, these stable QTL could play an important role in improving fiber quality and yield traits.

### Genetic effect and interaction of the introgressed chromosome segments

Clustered distribution of QTL is a relatively common phenomenon [38–41]. Said et al. [42] analyzed 2,134 previously reported QTL in intra- and inter-species populations and found numerous QTL that were distributed in clusters within defined chromosome regions in the specific populations. We found some clusters on the introgressed segments of the cotton genomes. Five QTL (qFL-5-1, qFM-5-1, qFS-5-1, qBW-5-1andqLP-5-1) were distributed in clusters linked to TMB1296 in Seg-5-1. Two QTL (qFL-7-1 and qFS-7-1) were linked to NAU1085 on Seg-7-1. Two QTL (qFM-8-1 and qFS-8-1) were linked to NAU1209 on Seg-8-1. Eleven QTL (3 for lint percentage, 5 for fiber strength, 1 for fiber length, boll weight and micronarie respectively) were distributed in clusters in the regions from 152.25 cM to 194 cM in chro20. Lacape etc. [43] suggested that these clustered QTL may belong to the same genetic factor group contributing to the complex network of fiber development and affecting the multiple fiber quality traits. Zhang et al. [44] reported that improvement of auxin expression level in epidermis of cotton ovule in the initial stage of fiber development could increase lint percentage and decrease fiber micronaire. The cluster on Seg-7-1 was identified by Guo [36]. The chromosome segments with these QTL hotspot or clusters could be useful for molecular breeding based on common molecular markers [42]. When the favorable alleles of the QTLs for cotton fiber quality and yield traits are clustered in the same chromosome segments they could be more easily used for simultaneous improvement of traits.

In addition to QTL, interactions are an important genetic basis for improvement of cotton yield and fiber quality traits [45, 46]. The interactions can be divided into three types (QTL with QTL, QTL with non-QTL, non-QTL with non-QTL) [47]. Therefore, the study of cotton molecular markers should be extended to non-mendelian factors [46].

A total of 5 QTL and 13 pairs of interactions for fiber length were detected on Seg-5-1, Seg-7-1, Seg-8-1, Seg-15-1, Seg-15-2, Seg-16-1, Seg-20-2, Seg-20-3 and Seg-22-1. Among them, interaction of different QTL were identified between Seg-5-1 with Seg-7-1 and Seg-5-1 with Seg-22-1. There are interactions of QTL by non-QTL between Seg-5-1 with Seg-8-1, Seg-5-1 with Seg-15, Seg-5-1 with Seg-20-2, Seg-7-1 with Seg-8-1, Seg-7-1 with Seg-15-1, Seg-7-1 with Seg-20-3, Seg-20-2 with Seg-20-3, and Seg-20-2 with Seg-22-1. Two interactions (Seg-8-1 with Seg-20-2 and Seg-15-2 with Seg-20-2,) were between non-QTL and non-QTL.

A total of 9 QTL and 13 pairs of interactions for fiber strength were detected on Seg-5-1, Seg-7-1, Seg-8-1, Seg-15-1, Seg-15-2, Seg-20-1, Seg-20-2, Seg-20-4 and Seg-22-1. A total of 8 interactions (Seg-5-1 with Seg-7-1, Seg-5-1 with Seg-20-2, Seg-7-1 with Seg-8-1, Seg-7-1 with Seg-20-2, Seg-7-1 with Seg-20-4, Seg-8-1(the two regions) with Seg-20-1 and Seg-20-1 with Seg-20-2) were between the different QTLs. A total of 4 interactions (Seg-7-1 with Seg-15-1, Seg-8-1 with Seg-15-1, Seg-15-1 with Seg-20-4, and Seg-15-2 with Seg-20-2) were between QTL and non-QTL. One interaction (Seg-15-2 with Seg-22-1) was between non-QTL and non-QTL.

A total of 3 pairs of interaction (Seg-7-1with Seg-8-1, Seg-8-1 with Seg-20-2, Seg-20-2 with Seg-20-3) for fiber length and 3 pairs of interaction (Seg-5-1 with Seg-20-2, Seg-7-1 with Seg-8-1, Seg-7-1 with Seg-15-1) for fiber strength could be identified in the two generations. The results indicate that the stable interactions should be considered for cotton breeding.

A total of 4 pairs of interaction (Seg-5-1 with area around 165 cM on Seg-20-2, Seg-7-1 with Seg-8-1, Seg-7-1 with Seg-15-1, Seg-15-2 with Seg-20-2) were found to affect fiber length and fiber strength simultaneously. These interactions could further explain the correlation between fiber length and fiber strength, and contribute to the simultaneous improvement of fiber length and fiber strength.

Comprehensive analysis of the QTL mapping and interaction of 19 *G*. *barbadense* cotton chromosome segments introgressed into CCRI36 indicated that five chromosome segments (Seg-5-1, Seg-7-1, Seg-8-1, Seg-20-2 and Seg-20-3) have important effects on cotton yield and fiber quality and merit further study.

Seg-5-1 was between BNL1042 and MGHES06 on chromosome 5. A total of 5 QTL (qLP-5-1, qBW-5-1, qFL-5-1, qFM-5-1 and qFS-5-1) were detected on this segment. The positive additive effects for qBW-5-1, qFL-5-1 and qFS-5-1 and the negative additive effects for qLP-5-1 and qFM-5-1 indicated that the segment can increase BW, FL and FS and decrease LP and FM. The homozygous segment could increase fiber length when interacting with Seg-7-1, Seg-8-1, Seg-22-1 and the region around 165 cM on Seg-20-2. The homozygous segment could increase fiber strength when interacting with Seg-8-1 and Seg-20-2.

Seg-7-1 was between NAU1085 and HAU1483 on chromosome 7. The positive additive effects of qFL-7-1 and qFS-7-1indicated that the segment can increase fiber length and fiber strength. The homozygous segment could increase fiber length interacting with Seg-5-1, Seg-8-1, Seg-15-1 and Seg-20-3. The homozygous segment could increase fiber strength interacting with Seg-8-1 and Seg-20-4.

Seg-8-1 was between CGR5759 and CICR0492 on chromosome 8. The positive additive effects for qFS-8-1 and the negative additive effects for qFM-8-1indicated that the segment can increase FS and decrease FM. The homozygous segment could increase fiber length interacting with Seg-5-1, Seg-7-1 and Seg-20-2. The homozygous segment could increase fiber strength interacting with Seg-5-1 and Seg-7-1.

Seg-20-2 was between NAU3813b and NAU4928 on chromosome 20. A total of 2 QTL clusters were detected on this segment. qLP-20-1 and qFS-20-2 were linked to NAU3813b (152.25 cM). The positive additive effects for qFS-20-2 and the negative additive effects for qLP-20-1indicated that the region around 152.25cM could increase FS and decrease LP. qLP-20-2, qBW-20-1 and qFS-20-3 were linked to TMB1125 (162.25 cM). The positive additive effects for qFS-20-3 and qLP-20-2 and the negative additive effects for qBW-20-1indicated that the region around 162.25 cM could increase FS and LP and decrease BW. The region around 152.25 cM on chromosome 20 could decrease fiber length interacting with Seg-5-1, and could increase fiber strength interacting with Seg-7-1. The homozygous fragment around 162.25 cM could increase FL interacted with Seg-20-3, and could increase fiber strength interacting with Seg-5-1.

Seg-20-3 was between NAU3665 and HAU0230b on chromosome 20. The positive additive effects for qLP-20-3, qFL-20-1, qFM-20-1, qFS-20-4 and qFS-20-5 indicated that the segment could increase LP, FL, FM and FS. The homozygous segment could increase FL interacting with Seg-7-1 and Seg-20-2.

These results show that fiber length and fiber strength both have a complex genetic basis that involves numerous interactions. Therefore, it is important to consider genetic interaction effects for fiber yield and quality in future research.

Chromosome segment substitution lines (CSSLs) are useful for the precise mapping of quantitative trait loci (QTLs) and dissection of the genetic basis of complex traits. Li et al. [48] confirmed a major QTL (qGR2) on chromosome 2 by using the CSSL-derived F_2_ population, and delimited to a 10.4 kb interval containing three putative candidate genes, of which OsMADS29 was only expressed preferentially in the seed. Functional analysis using CSSLs with HI6 indicated that HI6 reduced the size of the lower parts of the plant, which is not important for production, while maintaining the size of the other organs related to production (e.g., flag leaf and panicle), resulting in improved nitrogen use efficiency[49]. Li et al. [50] precisely map qRBSDV-6MH to the markers S18 and S23 at a physical distance of 627.6 kb on the Nipponbare reference genome using a set of chromosome segment substitution lines. Liu et al.[51] mapped SPP1 to a 2.2-cM interval between RM1195 and RM490 using a random NIL-F2 population of 210 individuals, which explained 51.1% of SPP variation. And then, four newly developed InDel markers were used for high-resolution mapping of SPP1 with a large NIL-F_2_ population. Finally, it was narrowed down to a bacterial artificial chromosome clone spanning 107 kb; 17 open reading frames have been identified in the region [51]. Liu et al. [52] mapped a major QTL influencing four fiber quality traits to a 0.28-cM interval and identified three candidate genes by RNA-Seq and RT-PCR analysis. Fang et al. [53] mapped qFS07.1 into a 62.6-kb genome region which contained four annotated genes on chromosome A07 of G. hirsutum. This study clearly showed that 5 chromosome segments (Seg-5-1, Seg-7-1, Seg-8-1, Seg-20-2 and Seg-20-3) have important effects on fiber yield and quality. The result provides the useful information for fine-mapping, gene cloning and marker-assisted breeding for excellent fiber related quality.

## Supporting information

S1 Table Interaction about fiber strength in two generation. (XLSX)

S2 Table Interaction about fiber length in two generation. (XLSX)

## References

[1] Huang ZK. Genetics and breeding of cotton in China. Jinan: Shandong Science and Technology Press, 2003.

[2] Qian N. Genetic diversity and association analysis of gene(QTL) of breeding target traits of upland cotton abstract. Nanjing: Nanjing Agricultural University, 2009

[3] Smith CW. Coyle GG. Association of fiber quality parameters and within-boll yield components in Upland cotton. Crop Sci. 1997; 37:1775–1779

[4] Liu JY, Zhao GR, Li J. Molecular engineering on quality improvement of cotton fiber. Acta Bot Sin.2000; 42(10):991–995

[5] Clement JD, Constable GA, Stiller WN, Liu SM.Negative associations still exist between yield and fiber quality in cotton breeding programs in Australia and USA. Field Crop Res.2012; 128(2), 1–7.

[6] Ma L, Su JJ, Chen H, and Deng FJ. Research progress of cotton molecular marker-assisted breeding, Guangdong Nongye Kexue (Guangdong Agri Sci).2014; 2: 138–143

[7] Reinisch A.J, Dong J.M, Brubaker C.L, Stelly D.M, Wendel J.F, and Paterson A.H. A detailed RFLP map of cotton,*Gossypium hirsutum ×Gossypium barbadense*: chromosomeorganization and evolution in a disomic polyploid genome,Genetics. 1994; 138: 829–847 7851778 10.1093/genetics/138.3.829 PMC1206231

[8] Jiang C X, Elziik K M, Paterson A H. Polyploid formation created unique avenues for response to selection in *Gossypium* (cotton). Pro Natl Acad Sci.1998; 95: 4419–4424 10.1073/pnas.95.8.4419 PMC22504 9539752

[9] Paterson AH, Saranga Y, Menz M, Jiang CX, Wright RJ. QTL analysis of genotype× environment interactions affecting cotton fiber quality. Theor Appl Genet.2003; 106(3): 384–396 doi: 10.1007/s00122-002-1025-y 12589538 10.1007/s00122-002-1025-y

[10] Wu MQ, Zhang XL, Nie YC, He DH. Localization of QTLs for yield and fiber quality traits of tetraploid cotton cultivar. Acta genet Sin. 2003; 30(5): 443–452 12924159

[11] Jamshed, Jia F, Gong JW, Koffi KP, Shi YZ, Li JW, et al Identification of stable quantitative trait loci(QTLs) for fiber quality traits across multiple environments in *Gossypium hirsutum* recombinant inbred line population.BMC Genomics. 2016; 17:197 doi: 10.1186/s12864-016-2560-2 26951621 10.1186/s12864-016-2560-2 PMC4782318

[12] Wang H, Huang C, Guo H, Li X, Zhao W, Dai B, et al QTL mapping for fiber and yield traits in upland cotton under multiple environments.PLoS ONE. 2015; 10(6): e0130742 doi: 10.1371/journal.pone.0130742 26110526 10.1371/journal.pone.0130742 PMC4481505

[13] Yang XL, Zhou XD, Wang XF, Li ZK, Zhang Y, Liu HW, et al Mapping QTL for cotton fiber quality traits using simple sequence repeat markers, conserved intron-scanning primers, and transcript-derived fragments. Euphytica. 2015; 201:215–230 doi: 10.1007/s10681-014-1194-1

[14] Wang P, Ding Z Y, Lu Q X, Guo W Z, Zhang T Z. Development of *Gossypium barbadense* chromosome segment substitution lines in the genetic standard line TM-1 of *Gossypium hirsutum*. Chinese Sci Bull. 2008; 53(9): 1065–1069

[15] Eshed Y, Zamir D. A genomic library of *Lycopersicon pennellii* in *L*.*esculentum*: A tool for fine mapping of genes. Euphytica.1994; 79: 175–179.

[16] Zhu WY, Lin J, Yang DW. Development of chromosome segment substitution lines derived from backcross between two sequenced rice cultivars, Indica Recipient 93–11 and Japonica Donor Nipponbare.Plant Mol Biol. 2009; 27: 126–131

[17] Szalma SJ, Hostert BM, LeDeaux JR, Stuber CW, Holland JB. QTL mapping with near-isogenic lines in maize. Theor Appl Genet. 2007; 114: 1211–1228. doi: 10.1007/s00122-007-0512-6 17308934 10.1007/s00122-007-0512-6

[18] Liu SB, Zhou RH, Dong YC, Li P, Jia JZ, et al Development utilization of introgression lines using a synthetie Wheat as donor.Theor Appl Genet.2006:112(7):1360–2373 doi: 10.1007/s00122-006-0238-x 16550399 10.1007/s00122-006-0238-x

[19] Stelly DM, Saha S, Raska DA, Jenkins JN, McCarty JC, Gutierrez OA.Registration of 17 Upland (*Gossypium hirsutum*) cotton germplasm lines disomic for different G.barbadense chromosomes or arm substitution. Crop Sci.2005; 45: 2663–2665.

[20] Luan M, Guo X, Zhang Y, Yao J, and Chen W. QTL mapping for agronomic and fiber traits using two inter specific chromosome substitution line s of upland cotton, Plant Breeding.2009;128(6): 671–679

[21] Zhu YJ, Wang P, Guo WZ Mapping QTLs for lint percentage and seed index using *Gossypium barbadense* chromosome segment introgression lines. Acta Agron Sin.2010; 36(8): 1318–1323.

[22] Wang P, Zhu YJ, Song XL, Cao ZB, Ding YZ, Liu BL, Zhu XF, Wang S, Guo WZ, Zhang TZ. Inheritance of long staple fiber quality traits of *Gossypium barbadense* in *G*. *hirsutum* background using CSILs. Theor Appl Genet. 2012; 124:1415–1428 doi: 10.1007/s00122-012-1797-7 22297564 10.1007/s00122-012-1797-7

[23] Cao ZB, Zhu XF, Chen H, Zhang TZ.Fine mapping of clustered quantitative trait loci for fiber quality on chromosome 7 using a *Gossypium barbadense* introgressed line. Mol Breeding. 2015; 35:215 doi: 10.1007/s11032-015-0393-3

[24] Wang F, Xu ZZ, Sun R, Gong YC, Liu GD, Zhang JX, et al Genetic dissection of the introgressive genomic components from Gossypium barbadense L. that contribute to improved fiber quality in Gossypium hirsutum L. Mol Breeding.2013; 32:547–562. doi: 10.1007/s11032-013-9888-y

[25] Shi YZ, Li WT, Li AG. Constructing a high-density linkage map for *Gossypium Hirsutum × G*. *barbadense* and identifying QTLs for lint percentage, J Integr Plant Biol. 2015; 57(5): 450–467. doi: 10.1111/jipb.12288 25263268 10.1111/jipb.12288

[26] Shi YZ, Zhang BC, Liu AY, Li WT, Li JW, Lu QW, et al Quantitative trait loci analysis of Verticillium wilt resistance in interspecific backcross populations of *Gossypium hirsutum ×Gossypium barbadense*.BMC Genomics. 2016; 17:877 doi: 10.1186/s12864-016-3128-x 27814678 10.1186/s12864-016-3128-x PMC5097350

[27] Liang Y, Jia YJ, Li AG, Zhang BC, Liu GP, Li JZ, et al Phenotyping traits related to yield and quality of BC5F2 substitution lines in cotton (*Gossypium*) and their QTL mapping. Mol Plant Breeding. 2010; 8(2):221–230.

[28] Li BT, Shi YZ, Gong JW, Li JW, Liu AY, Shang HH, et al Genetic Effects and Heterosis of Yield and Yield Component Traits Based on *Gossypium Barbadense* Chromosome Segment Substitution Lines in Two *Gossypium Hirsutum* Backgrounds.PLoS ONE. 2016; 11(6): e0157978 doi: 10.1371/journal.pone.0157978 27348815 10.1371/journal.pone.0157978 PMC4922568

[29] Zhai HC, Gong WK, Tan YN, Liu AY, Song WW, Li JW, et al Identification of chromosome segment substitution lines of *Gossypium barbadense* introgressed in G. hirsutum and quantitative trait locus mapping for fiber quality and yield traits. PLoS ONE. 2016; 11(9): e0159101 doi: 10.1371/journal.pone.0159101 27603312 10.1371/journal.pone.0159101 PMC5014324

[30] Zhang JF, Shi YZ, Liang Y, Jia YJ, Zhang BC, Li JW, et al Evaluation of yield and fiber quality traits of chromosome segment substitution lines population (BC_5_ F_3_ and BC_5_ F_3_:_4_) in cotton. J Plant Genet Resour. 2012; 13(5): 773–781.

[31] Paterson AH, Brubaker CL, Wendel JF. A rapid method for extraction of cotton (*Gossypium spp*.)genomic DNA suitable for RFLP or PCR analysis. Plant Mol Biol Rep. 1993;11: 122–127

[32] Zhang J, Wu YT, Guo WZ, Zhang TZ. Fast screening of microsatellite markers in cotton with PAGE/silver staining. Acta Gossypii Sin. 2000; 12(5): 267–269.

[33] Van B R. GGT 2.0: versatile software for visualization and analysis of genetic data. J Hered. 2008; 99: 232–236. doi: 10.1093/jhered/esm109 18222930 10.1093/jhered/esm109

[34] Wang J K, Wan X Y, Crossa J. QTL mapping of grain length in rice (Oryza sativa L.) using chromosome segment substitution lines. Genet Res. 2006; 88: 93–104. doi: 10.1017/S0016672306008408 17125584 10.1017/S0016672306008408

[35] Ma LJ.The evaluation and QTL Identifying of Chromosome Segment Substitution Lines in advanced backcross of *Gossypium hirsutum* × *G*. *barbadense*. Beijing: Chinese Academy of Agricultural Sciences,2015

[36] Guo LX.Genetic Analysis of the fiber quality traits in the segregting population of chromosome segment substitution lines from *Gossypium hirsutum×Gossypium barbadense*. Xinjiang: Shihezi University,2015

[37] He R.The evaluation and identifying QTL of chromosome segment substitution lines (BC_5_F_3_, BC_5_F_3:4_, BC_5_F_3:5_)in CCIR36 backgtound of *Gossypium hirsutum L*. Chongqing: Southwest University,2014

[38] Zhang ZS, Hu MC, Zhang J, Liu DJ, Zheng J, Zhang K, Wang W, Wan Q. Construction of a comprehensive PCR-based marker linkage map and QTL mapping for fiber quality traits in upland cotton (Gossypium hirsutum L.). Mol Breed. 2009; 24:49–61

[39] Tang SY, Teng ZZ, Zhai TF, Fang XM, Liu F, Liu DJ. Construction of genetic map and QTL analysis of fiber quality traits for Upland cotton (Gossypium hirsutum L.). Euphytica. 2015; 201:195–213

[40] Ning ZY, Chen H, Mei HX, Zhang TZ. Molecular tagging of qtls for fiber quality and yield in the upland cotton cultivar acala-prema. Euphytica. 2014; 195, 143–156.

[41] Wang HT, Huang C, Guo HL, Li XM, Zhao WX, Dai BS. QTL Mapping for fiber and yield traits in upland cotton under multiple environments. PLoS ONE. 2015; 10(6): e0130742 doi: 10.1371/journal.pone.0130742 26110526 10.1371/journal.pone.0130742 PMC4481505

[42] Said JI, Song MZ, Wang HT, Lin ZX, Zhang XL, Fang DD. A comparative meta-analysis of QTL between intraspecific *Gossypium hirsutum* and interspecific *G*. *hirsutum × G*. *barbadense* populations. Mol Genet Genomics. 2015; 290: 1003–1025. doi: 10.1007/s00438-014-0963-9 25501533 10.1007/s00438-014-0963-9

[43] Lacape JM, Llewellyn D, Jacobs J, Arioli T, Becker D, Calhoun S, et al Meta-analysis of cotton fiber quality QTLs across diverse environments in a *Gossypium hirsutum x G*.*barbadense* RIL population.BMC Plant Biol. 2010; 10:132: doi: 10.1186/1471-2229-10-132 20584292 10.1186/1471-2229-10-132 PMC3017793

[44] Zhang ZS, Rong JK, Waghmare VN, Chee PW, May OL, Wright RJ, et al QTL alleles for improved fiber quality from a wild Hawaiian cotton, *Gossypium tomentosum*. Theor Appl Genet, 2011;123:1075–1088 doi: 10.1007/s00122-011-1649-x 21735234 10.1007/s00122-011-1649-x

[45] Lin ZX, Feng CH, Guo XP, Zhang XL.Genetic analysis of major QTLs and epistasis interaction for yield and fiber quality in upland cotton. Scientia Agri Sin 2009; 42(9):3036–3047

[46] He DH, Lin ZX, Zhang XL, Nie YC, Guo XP.Dissection of genetic bases of fiber quality in *Gossypium hirsutum* with molecular markers. Cotton Sci. 2004: 16(3): 131~136

[47] Liao CY, Wu P, Hu B, Yi KK. Effects of genetic background and environment on QTLs and epistasis for rice (Oryza sativa L.) panicle number. Theor Appl Genet, 2001; 103: 104–111

[48] Li M, Sun PL, Zhou HJ, Chen S, Yu SB. Identification of quantitative trait loci associated with germination using chromosome segment substitution lines of rice (*Oryza sativa* L.). Theor Appl Genet 2011: 123:411–420 doi: 10.1007/s00122-011-1593-9 21512773 10.1007/s00122-011-1593-9

[49] Kazuhiro U, Takayuki K, Ken I. Identification and functional analysis of alleles for productivity in two sets of chromosome segment substitution lines of rice. Euphytica 2012: 187:325–337 doi: 10.1007/s10681-012-0660-x

[50] Li AH, Pan CH, Wu LB, Dai ZY, Zuo SM ea al Identification and fine mapping of qRBSDV-6^MH^, a major QTL for resistance to rice black-streaked dwarf virus disease. Mol Breeding. 2013: 32:1–13 doi: 10.1007/s11032-012-9807-7

[51] Liu TM, Mao DH, Zhang SP, Zu CG, Xing YZ. Fine mapping SPP1, a QTL controlling the number of spikelets per panicle, to a BAC clone in rice (*Oryza sativa*). Theor Appl Genet 2009: 118(8): 1509–1517 doi: 10.1007/s00122-009-0999-0 19266175 10.1007/s00122-009-0999-0

[52] Liu DX, Zhang J, Liu XY, Wang WW, Liu DJ, et al Fine mapping and RNA-Seq unravels candidate genes for a major QTL controlling multiple fiber quality traits at the T1 region in upland cotton. BMC Genomics 2016: 17:295 doi: 10.1186/s12864-016-2605-6 27094760 10.1186/s12864-016-2605-6 PMC4837631

[53] Fang XM, Liu XY, Wang XQ, Wang WW, Liu DX, et al Fine-mapping qFS07.1 controlling fiber strength in upland cotton (*Gossypium hirsutum* L.). Theor Appl Genet 2017: 130(4): 795–806 doi: 10.1007/s00122-017-2852-1 28144698 10.1007/s00122-017-2852-1

